# Control of the microsporidian parasite *Nosema ceranae* in honey bees (*Apis mellifera*) using nutraceutical and immuno-stimulatory compounds

**DOI:** 10.1371/journal.pone.0227484

**Published:** 2020-01-10

**Authors:** Daniel Borges, Ernesto Guzman-Novoa, Paul H. Goodwin

**Affiliations:** School of Environmental Sciences, University of Guelph, Guelph, Ontario, Canada; University of North Carolina at Greensboro, UNITED STATES

## Abstract

*Nosema ceranae* is a microsporidian parasite that causes nosemosis in the honey bee (*Apis mellifera*). As alternatives to the antibiotic fumagillin, ten nutraceuticals (oregano oil, thymol, carvacrol, *trans*-cinnmaldehyde, tetrahydrocurcumin, sulforaphane, naringenin, embelin, allyl sulfide, hydroxytyrosol) and two immuno-stimulatory compounds (chitosan, poly I:C) were examined for controlling *N*. *ceranae* infections. Caged bees were inoculated with *N*. *ceranae* spores, and treatments were administered in sugar syrup. Only two compounds did not significantly reduce *N*. *ceranae* spore counts compared to the infected positive control, but the most effective were sulforaphane from cruciferous vegetables, carvacrol from oregano oil, and naringenin from citrus fruit. When tested at several concentrations, the highest sulforaphane concentration reduced spore counts by 100%, but also caused 100% bee mortality. For carvacrol, the maximum reduction in spore counts was 57% with an intermediate concentration and the maximum bee mortality was 23% with the highest concentration. For naringenin, the maximum reduction in spore counts was 64% with the highest concentration, and the maximum bee mortality was only 15% with an intermediate concentration. In the longevity experiment, naringenin-fed bees lived as long as *Nosema*-free control bees, both of which lived significantly longer than infected positive control bees. While its antimicrobial properties may be promising, reducing sulforaphane toxicity to bees is necessary before it can be considered as a candidate for controlling *N*. *ceranae*. Although further work on formulation is needed with naringenin, its effect on extending longevity in infected bees may give it an additional value as a potential additive for bee feed in honey bee colonies.

## Introduction

Microsporidia are fungi that exist as obligate intracellular parasites of many invertebrate and vertebrate species, including insects [1]. The honey bee, *Apis mellifera*, is infected by two species of microsporidia, *Nosema apis* and *Nosema ceranae* [2]. Both *N*. *ceranae* and *N*. *apis* infect the midgut epithelium of *A*. *mellifera*, resulting in impaired digestion and absorption of nutrients and affecting metabolic processes. These microsporidia down-regulate genes related to intestinal health, nutrient absorption and antioxidant activity [3–5]. However, in places where both parasites exist, such as in Canada, USA and Europe, infections by *N*. *ceranae* have become more common than infections by *N*. *apis* [6–8]. *N*. *ceranae* causes suppression of the immune response in honey bees by downregulating the expression of antimicrobial peptide (AMP) genes [9], although another study showed that *N*. *apis* and *N*. *ceranae* can also up-regulate AMP genes of honey bees [10]. In addition, *N*. *ceranae* infections cause increased energetic stress on the bee, leading to degeneration of epithelial cells and significantly shortening its life span [3, 11]. Infection with *N*. *ceranae* has been associated with the loss of honey bee colonies in Europe and North America [12–15].

The only registered treatment for *N*. *ceranae* and *N*. *apis* infection for over 60 years has been the antibiotic bicyclohexylammonium fumagillin (fumagillin), isolated from the fungus *Aspergillus fumigatus* [16]. Although it degrades quickly in the hive, fumagillin residues can persist in honey and wax for up to six months [17]. These low concentrations of fumagillin can lead to the development of resistance, causing hyper-proliferation of *N*. *ceranae*, but not *N*. *apis* [18]. The development of fumagillin resistance in *N*. *ceranae* and the risk of contaminating honey with toxic residues point to a need for safer, alternative treatments for nosema disease.

An alternative approach to control intestinal diseases is the use of nutraceuticals, which are foods (or parts of foods) having health benefits, including the prevention and/or treatment of diseases through their antimicrobial, antioxidant, immuno-stimulatory and/or anti-inflammatory activities [19]. Examples of nutraceuticals with both antimicrobial and anti-inflammatory activities are (di)allyl sulfide, from garlic, that lowered infection prevalence of *Nosema bombycis* when given orally to the silkworm, *Bombyx mori* [20] and attenuated intestinal damage when fed to rats [21], and trans-cinnamaldehyde, from *Cinnamomum* spp. (cinnamon), which when fed to chickens, reduced intestinal populations of pathogenic bacteria [22] and down-regulated genes that induce inflammation [23]. Others have both antimicrobial and antioxidant activities, such as sulforaphane, from cruciferous vegetables, which increased expression of antioxidant genes when fed to mice [24] and suppressed growth of numerous bacteria and fungi *in vitro* [25]. Nutraceuticals with both anti-inflammatory and antioxidant activities include naringenin, a flavone from citrus fruit, that decreased expression of pro-inflammatory cytokines [26] and reduced oxidative damage when fed to mice [27] and rats [28], and the phenolic compound, hydroxytyrosol, from olive oil, that directly scavenged reactive oxygen species (ROS) and free radicals *in vitro* and in mice [29], while also down-regulating genes associated with inflammation in rats [30]. The hydroxyl benzoquinone, embelin, from *Embelia ribes* (false black pepper), also has anti-inflammatory and antioxidant properties, reducing the expression of pro-inflammatory cytokines in mice intestines [31], and increasing the expression of antioxidant genes when fed to rats [32]. Some nutraceuticals have immuno-stimulatory activity, such as the synthetic, double-stranded viral RNA molecule, polyinosinic:polycytidylic acid (poly I:C), which induced an immune response in chickens [33], and also induced a moderate immune response in the Pacific white shrimp, *Litopenaeus vannamei*, similar to a challenge with the pathogen, *Staphylococcus aureus* [34], and the acetylated chitin polysaccharide, chitosan, from the shells of crustaceans, that caused an increase in serum levels of a number of immunoglobulins when fed to chickens [35], and also increased expression of a number of AMPs when fed to honey bees [36].

Thus far, there have been relatively few attempts to use nutraceuticals to control *N*. *ceranae* and *N*. *apis* in honey bees. Feeding bees thyme and winter savory extracts containing the antimicrobial compounds, thymol and carvacrol (both of which are also found in oregano oil), in sugar syrup, did not reduce *Nosema* spp. spore loads in the field, although they did decrease bee mortality [37]. However, many other studies that fed bees thymol or the anti-inflammatory compound, resveratrol, found that both reduced *N*. *ceranae* and *N*. *apis* spore loads in infected bees, and resveratrol also decreased bee mortality [38, 39]. Similarly, feeding bees curcumin, an antimicrobial compound from turmeric (*Curcuma longa*), reduced *Nosema* spp. spore loads and increased survival of infected bees [40]. While they were not tested in this study, promising results have also been seen with algal polysaccharides [41], oxalic acid [42] and porphyrins [43], all of which reduced *N*. *ceranae* spore loads when fed to bees in sugar syrup.

The limited studies thus far indicate that nutraceuticals may be effective in controlling *N*. *ceranae* infection due either to antimicrobial properties against the parasite, anti-inflammatory and antioxidant properties to reduce symptoms of infection, or immuno-stimulant properties that may help the bee to fight the parasite. They may also increase longevity in infected bees. This study was conducted to screen a diverse collection of nutraceuticals and immuno-stimulants using caged bees to examine their potential in reducing *N*. *ceranae* spore counts and increasing the life span of inoculated honey bees.

## Materials and methods

### *Nosema ceranae* spore extraction

Honey bee foragers were collected from hives at the University of Guelph’s Honey Bee Research Centre in Ontario, Canada, using a modified vacuum [44]. Detection and quantification of *Nosema* spores were performed by microscopy as per Cantwell [45] on all samples to determine which colonies were highly infected. Bees collected from the most infected colonies were pooled and stored at -20 °C until spores were extracted.

For spore extraction, around 12 to 15 bee abdomens were crushed using a mortar and pestle and 25 ml of dH_2_O. The macerate was filtered using a piece of nylon honey filter with a pore size of 177 μm (Better Bee Supplies, Cambridge, Ontario, CA) before being centrifuged at room temperature for 8 min at 800 x g. The supernatant was discarded, and the remaining macerate was combined into a 2 ml tube and vortexed for 10 s. DNA was extracted from the spores and the presence of only *N*. *ceranae* was confirmed by PCR analysis as per Hamiduzzaman et al. [46].

### Inoculation with *N*. *ceranae* spores

Infection of honey bees with *N*. *ceranae* spores was done as per Maistrello et al. [37], with some modifications. Briefly, frames with capped brood from hives without detectable *Nosema* infection were incubated (35°C, 60% RH) overnight, and newly-emerged adult bees were collected in the morning. Bees were starved for 2 h before inoculation, and *Nosema* spore diagnosis was performed on a sample of 15 bees to ensure that the newly-emerged bees had no detectable *Nosema* spores. Extracted spores were quantified using a haemocytometer and diluted to 10,000 spores/μl in 50% sugar syrup. Bees were individually fed 5 μl of the sugar syrup containing the extracted spores using a micropipette (Eppendorf, Mississauga, Ontario, CA) with each bee receiving approximately 50,000 spores, which ensures infection of >98% of individual bees [47]. Bees that did not consume the entire 5 μl of inoculum were discarded. After feeding, batches of 40 bees were placed in wooden hoarding cages (13.0 x 9.5 x 15 cm), and maintained in an incubator at 33°C and 65% RH. Negative control bees were individually fed 5 μl of sugar syrup without spores.

### Treatments

The nutraceuticals oregano oil, carvacrol, thymol, allyl sulfide, trans-cinnamaldehyde, and (±)-naringenin, and the immuno-stimulatory compound poly I:C sodium salt were obtained from Sigma-Aldrich (Oakville, Ontario, CA). The nutraceuticals D,L-sulforaphane, hydroxytyrosol, embelin, and tetrahydrocurcumin were obtained from Toronto Research Chemicals Inc. (Toronto, Ontario, CA). The immune-stimulatory compound polyglucosamine (low molecular weight chitosan) was obtained from BioAmber Inc. (DNP Green Technology, Montreal, Quebec, CA). Fumagillin-B was obtained from Medivet Pharmaceuticals Ltd. (High River, Alberta, CA). Concentrations were determined by examining studies in other organisms (Table 1). When the compound was administered as a dose per unit body weight to animals other than honey bees in those studies, the appropriate dose per bee was calculated based on an average body weight of 100 mg per honey bee, and the total dose for 40 bees was added to the sugar syrup each time feeders were changed. The reported concentration for this study was calculated based on this dose for 40 bees and the amount of sugar syrup in the feeder. When the studies listed in Table 1 administered the compound at a particular concentration instead of a dose per unit body weight, that concentration was used for this study. Concentrations used in this study (in mg/ml of sugar syrup) are shown in Table 1.

**Table 1:** Concentrations of fumagillin and the compounds used for the screening experiment, and the source for each concentration. The reported concentrations were either calculated from the study doses using an average body weight of 100 mg per honey bee, or they are the same as the concentration used in the study cited. All concentrations are listed in mg/ml of 50% sugar syrup.

Treatment	Concentration (mg/ml)	Study	Method	Species
Fumagillin	0.0500^1^	[48]	Feed	Honey bees
Oregano oil	0.1250	[49]	Feed	Chickens
Thymol	0.1250	[38]	Feed	Honey bees
Carvacrol	0.1000	[50]	Feed	Mice
Chitosan	0.0600	[35]	Feed	Chickens
*trans*-cinnamaldehyde	0.1000	[51]	Feed	Hamsters
Tetrahydrocurcumin	0.2000	[52]	Feed	Rats
Sulforaphane	0.1667	[24]	Feed	Mice
Naringenin	0.1000	[26]	Feed	Mice
Embelin	0.1000	[31]	Feed	Mice
Allyl sulfide	0.0300	[20]	Feed	Rats
Hydroxytyrosol	0.2000	[30]	Feed	Rats
Poly I:C	0.0183	[53]	Injection	Chickens

^1^ Fumidil-B powder contains 21 mg of fumagillin/g of powder; 2.3810 mg/ml Fumidil-B powder was used, containing the appropriate concentration of 0.0500 mg/ml fumagillin.

When determining concentrations for oregano oil, thymol, and carvacrol, oral toxicity in honey bees was also considered [54, 55].

### Primary screening

Each cage of 40 bees was administered one compound mixed in 50% sugar syrup in 15 ml drip feeders immediately after being caged and inoculated. Ethanol at a concentration of 4 μl/ml of sugar syrup was added to feeders containing oregano oil, thymol, carvacrol, fumagillin, tetrahydrocurcumin, sulforaphane, naringenin, embelin, and allyl sulfide to aid in dissolving the compounds. The feeders containing chitosan, trans-cinnamaldehyde, poly I:C, and hydroxytyrosol had the same volume of distilled water added instead of ethanol to aid in dissolving the compounds. No phase separation was observed with any of the compounds. Non-inoculated, negative control and inoculated, positive control bees, were both given drip feeders containing only 50% sugar syrup. The standard treatment control consisted of inoculated bees fed sugar syrup containing 0.05 mg/ml fumagillin. Feeders containing water were also provided for all cages. Feeders were changed every four days and weighed before and after changing using a balance (Model S-403, Denver Instrument, Bohemia, New York, USA) to determine syrup consumption. The average number of bees alive between each feeder change and the amount of syrup consumed was used to estimate total feed intake per bee over 16 days. Dead bees were removed daily and counted. At 16 days post-inoculation (dpi), remaining bees were sacrificed and stored at -20°C. Spore counts were done as per Cantwell [45] using the midguts of the remaining bees as one pooled sample per cage. Bee mortality was calculated as percent mortality over a period of 16 dpi excluding bees that died within 2 dpi, as this is typically due to handling and inoculation stress and not *N*. *ceranae* infection; it never exceeded three bees in all cases. The experiment was replicated three times for a total of 45 cages (12 compounds + 3 control treatments x 3 replicates).

### Dose responses with sulforaphane, carvacrol and naringenin

Dose response relationships for spore count, bee mortality and feed intake were determined using the above procedures for 0, 0.0125, 0.1250, 0.1667, 0.6250 and 1.2500 mg/ml sulforaphane, 0, 0.0125, 0.1000, 0.1250, 0.6250 and 1.2500 mg/ml carvacrol and 0, 0.0208, 0.1000, 0.2083, 1.0417 and 2.0833 mg/ml naringenin. The concentrations were calculated based on the results of the screening experiment. The experiment was replicated three times.

### Honey bee longevity with sulforaphane and naringenin

To assess long-term survival and mortality, treatments were done with 0.2917 mg/ml sulforaphane and 4.1667 mg/ml naringenin following the above procedures, except that 30 bees were used per cage instead of 40, and the experiment was allowed to continue until every bee had died rather than at 16 dpi. Mortality was measured as previously described, but bees were not sacrificed at any point for spore counts. Instead, spore counts were performed at 10 and 15 dpi on samples of individual bees that had died on those days (3–10 bees), to ensure normal pathogen development. Cumulative survival was derived from the mortality data and the experiment was repeated three times. Kaplan-Meier survival curves were created for each treatment using the cumulative survival data for individual bees within a particular treatment cage [56]. As with previous experiments, bees that died within 2 dpi were excluded from the analysis.

### Statistical analysis

An analysis of variance with the General Linear Model (GLM) procedure at p = 0.05 was used to determine differences among the treatments. When significant treatment effects were found, means were separated by Least Significant Difference tests (LSD, p = 0.05). Best fit regression models were used to examine the relationship between spore counts, mortality and feed intake to different concentrations of selected treatments. As responses to concentrations were pooled from the primary screening and dose response experiments, the positive control values were pooled for the analyses. Because of the low number of bees alive for much of the experiment with 0.625 mg/ml sulforaphane, the feed and water intake appeared to be over-estimated due to normal dripping of the sugar syrup and water feeders, and thus the feed intake and water intake values for that concentration were excluded from all analyses. The honey bee longevity experiment was analysed by creating Kaplan-Meier survival curves for the bees in each treatment. The curves were compared using a log-rank/Mantel-Cox post hoc test to determine which curves were significantly different from one another. All statistical analyses were conducted using SPSS version 22 (IBM SPSS Statistics, Armonk, New York), and all tests used a Type I error rate of 0.05 to determine significance.

## Results

### Primary screening

No *Nosema* spores were detected in the negative control and fumagillin treatments, while the highest spore counts were for the positive control (Table 2). Based on the spore counts, all the treatments were significantly different from the positive control, except for hydroxytyrosol and trans-cinnamaldehyde (p < 0.0001). The greatest reduction in spore counts was 64.0% for sulforaphane, although this was not significantly different from the spore counts with carvacrol, naringenin, tetrahydrocurcumin, thymol, oregano oil or embelin (Table 2).

**Table 2:** Mean *N*. *ceranae* spore counts per bee ± SE of infected honey bees fed different compounds. Treatments followed by the same letter are not significantly different based on ANOVA and Fisher’s LSD tests (α = 0.05).

Treatment	Mean spore count (spores/bee±SE)	Mean percent reduction (±SE)^1^	Means comparison
Negative control	0.00 ± 0.00	100 ± 0.00	a
Fumagillin	0.00 ± 0.00	100 ± 0.00	a
Sulforaphane	7.71E+06 ± 7.96E+05	64.0 ± 3.36	b
Carvacrol	9.16E+06 ± 2.02E+06	56.7 ± 10.1	b, c
Naringenin	10.9E+06 ± 1.61E+06	49.0 ± 7.09	b, c, d
Tetrahydrocurcumin	11.3E+06 ± 1.58E+06	47.1 ± 6.58	b, c, d
Thymol	12.8E+06 ± 1.81E+06	40.6 ± 7.31	b, c, d
Oregano oil	13.1E+06 ± 4.89E+06	39.6 ± 22.2	b, c, d
Embelin	13.1E+06 ± 3.04E+06	37.7 ± 12.9	b, c, d
Allyl sulfide	14.2E+06 ± 9.52E+05	33.2 ± 5.84	c, d
Chitosan	14.9E+06 ± 2.41E+06	29.9 ± 13.0	c, d
Poly I:C	15.0E+06 ± 2.62E+06	29.8 ± 12.0	c, d
Hydroxytyrosol	15.1E+06 ± 9.96E+05	28.9 ± 1.94	c, d, e
cinnamaldehyde	16.2E+06 ± 2.74E+06	24.4 ± 12.0	d, e
Positive control	21.3E+06 ± 4.81E+05	0.00 ± 0.00	e

^1^ Mean percent reduction was calculated based on the spore count for the positive control for each replicate and then averaged.

While bee mortality was higher in the positive control than in the negative control, there were no significant differences between the controls or any of the treatments for bee mortality (p > 0.05; Table 3). Feed intake also showed no significant differences between the controls or any of the treatments, although it was only notably lower with fumagillin treatment (p > 0.05; Table 3).

**Table 3:** Mean bee mortality ± SE (%) and mean feed intake ± SE (mg of syrup/bee over 16 days) of *N*. *ceranae*-infected honey bees fed different compounds. No significant differences were found between treatments for bee mortality or feed intake using an ANOVA (α = 0.05).

Treatment	Mean bee mortality (%)^1^	Mean feed intake (mg of syrup/bee over 16 days)
Positive control	7.12 ± 4.79	591.2 ± 40.19
Negative control	4.27 ± 4.27	554.9 ± 49.53
Fumagillin	18.5 ± 9.24	389.6 ± 77.95
Sulforaphane	23.2 ± 7.55	403.9 ± 33.70
Carvacrol	13.6 ± 1.83	503.9 ± 35.37
Naringenin	9.34 ± 4.60	548.0 ± 10.58
Tetrahydrocurcumin	15.7 ± 8.14	536.7 ± 31.39
Thymol	12.8 ± 5.57	425.2 ± 34.02
Oregano oil	15.7 ± 4.13	450.8 ± 48.84
Embelin	7.69 ± 5.13	565.4 ± 72.42
Allyl sulfide	13.0 ± 6.13	566.1 ± 45.13
Chitosan	12.2 ± 6.17	548.4 ± 48.08
Poly I:C	9.36 ± 2.17	513.0 ± 54.44
Hydroxytyrosol	9.83 ± 1.94	512.5^2^
cinnamaldehyde	7.64 ± 5.28	585.1 ± 95.75

^1^ Bees that died within 2 dpi were excluded from the mortality calculations.

^2^ Not an average as feeder weights were lost (feeders leaked out completely); as it is not an average, no standard error could be calculated; value is from replicate 3.

The three treatments showing the greatest reduction in spore counts, sulforaphane, carvacrol and naringenin, in the primary screening (Table 2), were further examined with a range of concentrations to determine their effectiveness in affecting spore counts, survivorship and feed intake.

### Relationship between *Nosema* spore counts and concentrations of sulforaphane, carvacrol and naringenin

**Fig 1:**
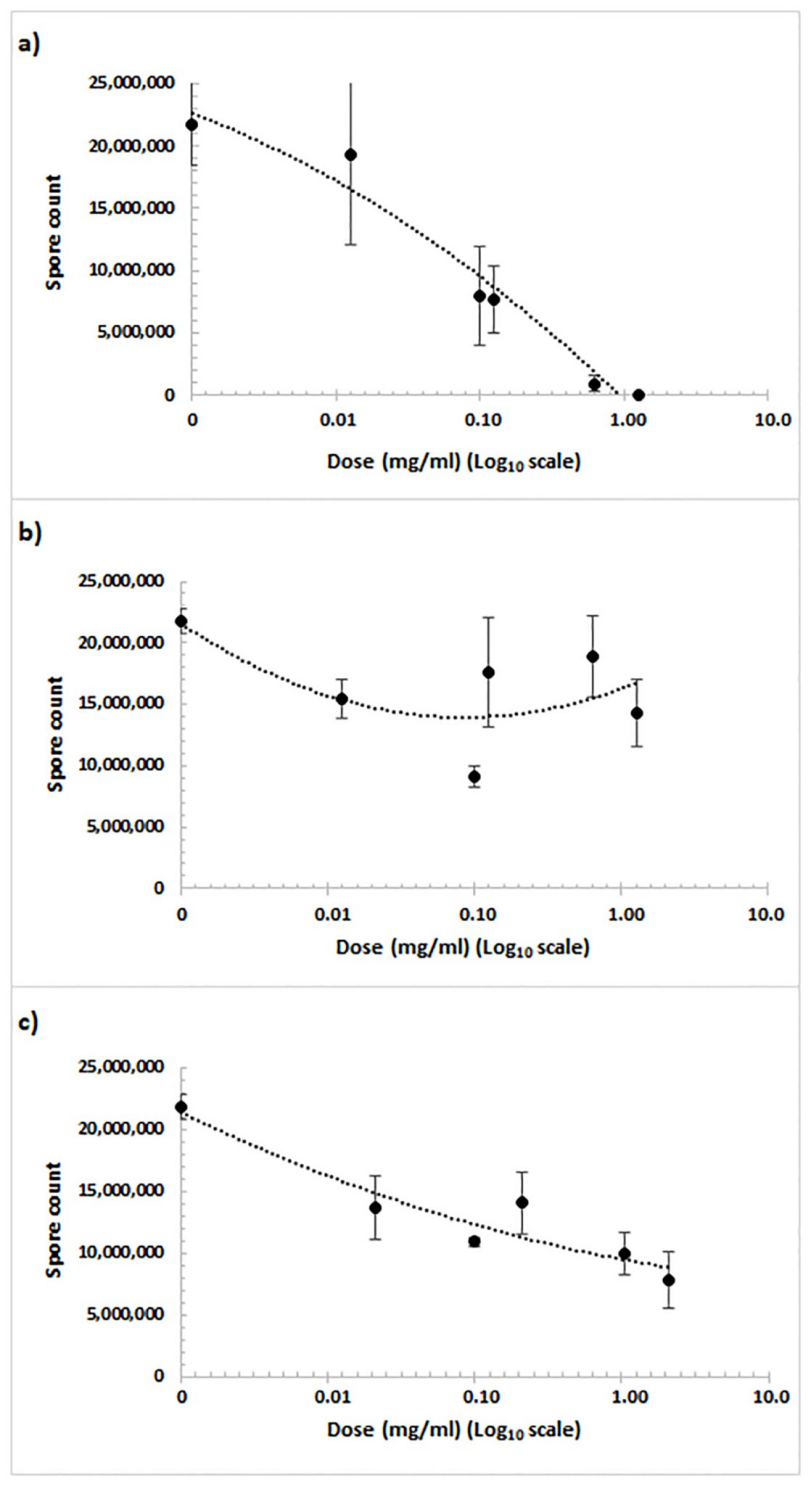
Relationship between spore count (number of spores/bee) on 16 dpi and log_10_ of the concentration of sulforaphane (a), carvacrol (b) and naringenin (c) for *N*. *ceranae*-infected honey bees.

A significant regression line for spore count versus the log of sulforaphane concentration was obtained (R^2^ = 0.9645, p < 0.0001, y = 1 X 10^6^x^2^–4 x 10^6^ x + 2 X 10^7^; Fig 1a). Except for the lowest concentration (0.0125 mg/ml), spore counts with all concentrations tested were significantly lower than that of the non-treated positive control (p < 0.05). The highest concentration of sulforaphane (1.2500 mg/ml) eliminated spores completely in all three replicates, and the next highest concentration (0.6250 mg/ml) reduced spore counts to around 9.2 x 10^5^ spores/bee, corresponding to a 95% reduction from the positive control. Spore counts with these two highest concentrations were significantly lower than spore counts with any other sulforaphane concentrations.

The regression for spore count vs. log of the carvacrol concentration was also significant (R^2^ = 0.4289, p <0.001, y = 2 X 10^6^x^2^ − 8 x 10^6^x + 2 X 10^7^; Fig 1b). The spore counts were significantly lower for all the concentrations tested compared to the positive control, except for the lowest concentration (0.0125 mg/ml; p < 0.05). The greatest reduction was for the second lowest concentration (0.1000 mg/ml) resulting in around 9.2 x 10^6^ spores/bee, corresponding to a 57% reduction from the positive control, which was significantly lower than the spore counts observed for any other concentrations of carvacrol.

The regression for spore count vs. log of the naringenin concentration was also significant (R^2^ = 0.68949, p <0.001, y = 5.8 X 10^5^x^2^ − 6 x 10^6^x+ 2 X 10^7^; Fig 1c), and spore counts for all concentrations tested were significantly lower than that of the non-treated positive control (p < 0.05). The highest concentration (2.0833 mg/ml) was the most effective in reducing spore counts, which were around 7.8 x 10^6^ spores/bee, corresponding to a 64% reduction from the positive control, but none of the spore counts with the different naringenin concentrations were significantly different from each other.

### Relationship between bee mortality and concentrations of sulforaphane, carvacrol and naringenin

**Fig 2:**
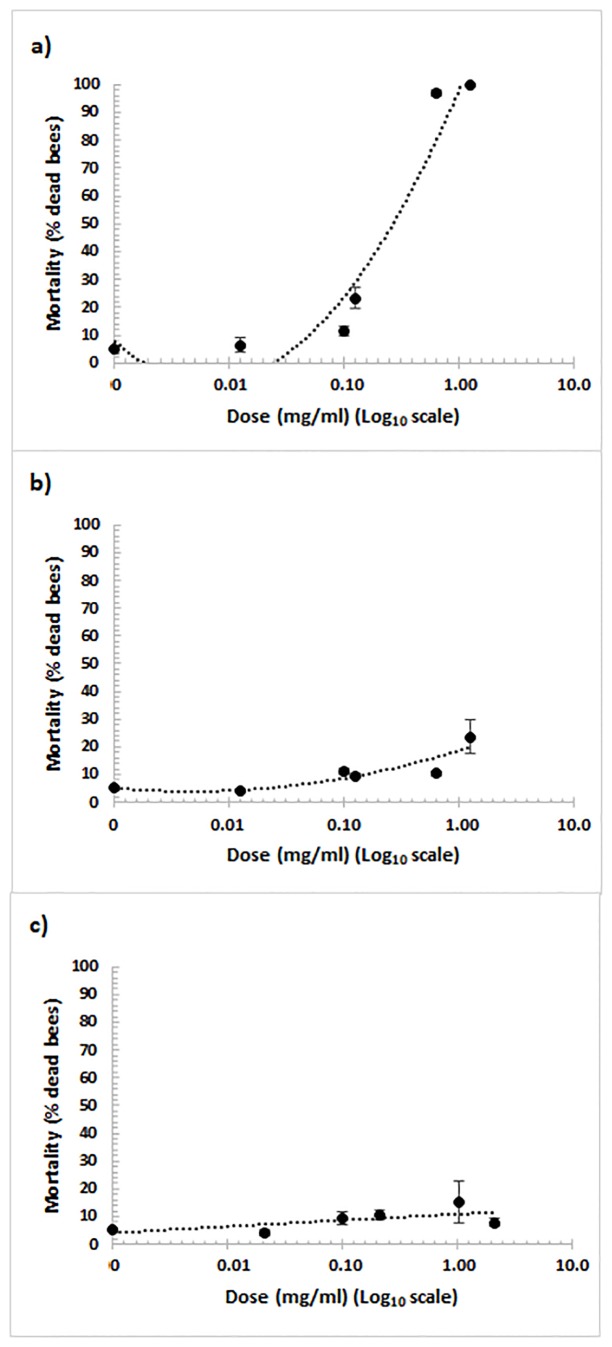
Relationship between percent bee mortality on 16 dpi and log_10_ of the concentration of sulforaphane (a), carvacrol (b) and naringenin (c) for *N*. *ceranae*-infected honey bees.

A significant regression line for mortality vs. the log sulforaphane concentration was obtained (R^2^ = 0.92325, p < 0.0001, y = 21.967x^2^ − 35.921x + 7.8738; Fig 2a) with mortality clearly increasing with concentration. All concentrations of sulforaphane tested caused significantly greater mortality than that observed for the non-treated positive control, except for the lowest concentration (0.0125 mg/ml; p < 0.05). The highest concentration of sulforaphane (1.2500 mg/ml) resulted in 100% bee mortality, and the next highest concentration (0.6250 mg/ml) caused 97% bee mortality, indicating high toxicity.

For carvacrol, a significant linear regression for mortality vs. log of the concentration was observed (R^2^ = 0.7812, p <0.0001, y = 2.7043x^2^ + 3.6795x + 5.2267; Fig 2b) with mortality generally increasing with concentration. However, even the highest average mortality with the highest concentration of carvacrol (1.2500 mg/ml) was relatively low at 23.52%. That was the only significant difference in bee mortality between the carvacrol concentrations or with the positive control (p <0.05), indicating low toxicity.

For naringenin, the regression line for mortality vs. log of the concentration was significant (R^2^ = 0.4477, p <0.0001, y = -0.0298x^2^ + 2.3462x + 4.1804; Fig 2c). Only the second highest concentration (1.0417 mg/ml) resulted in significantly different bee mortality compared to the other concentrations or the positive control (p <0.05). However, naringenin appeared to have low toxicity as even the highest bee mortality was only 15% at that concentration.

### Relationship between feed intake and concentrations of sulforaphane, carvacrol and naringenin

**Fig 3:**
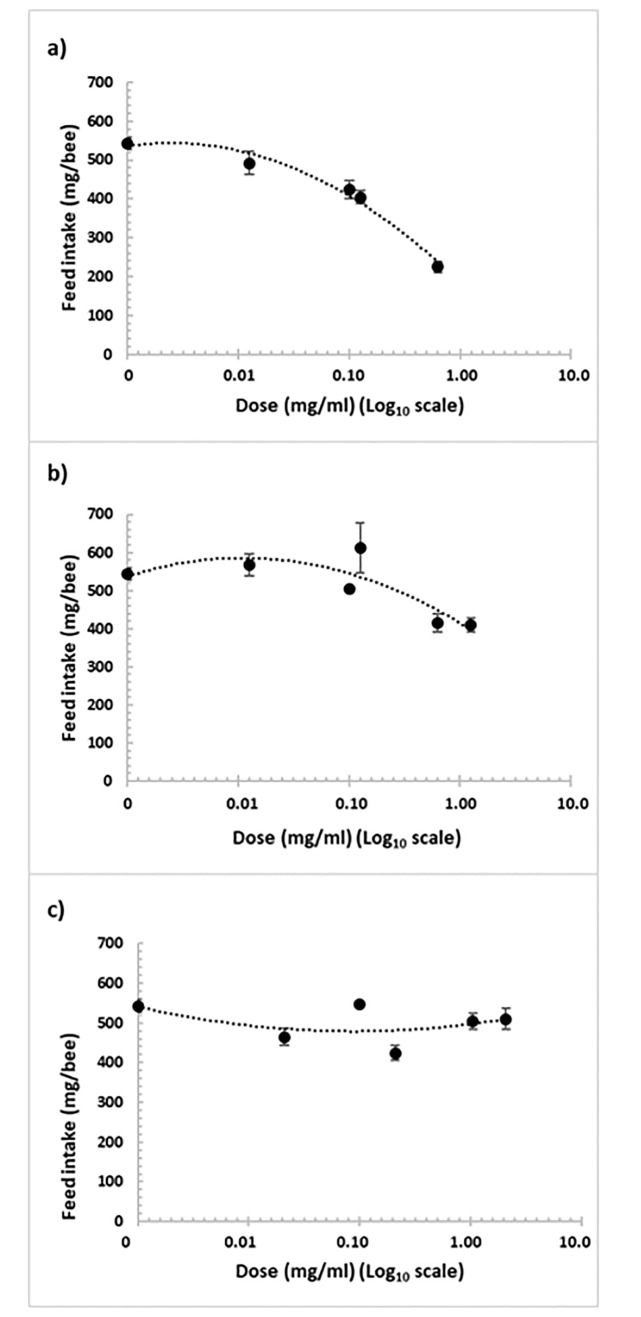
Relationship between feed intake (mg syrup/bee over 16 days) and log_10_ of the concentration of sulforaphane (a), carvacrol (b) and naringenin (c) for *N*. *ceranae*-infected honey bees.

For feed intake, a significant regression line for the log concentration of sulforaphane was observed (R^2^ = 0.217, p < 0.0001, y = -12.975x^2^–12.933x + 532.28; Fig 3a). Feed intake was significantly lower than the positive control for all the concentrations tested, except for the lowest concentration (0.0125 mg/ml; p < 0.05). The lowest feed intake (225.17 mg syrup/bee) was seen with the highest concentration of sulforaphane (1.2500 mg/ml), which showed a 58% reduction from that of the positive control.

For carvacrol, the regression line for feed intake vs. log of the concentration was significant (R^2^ = 0.733, p <0.0001, y = -44.326x^2^ + 92.299x + 537.69; Fig 3b). However, the highest (1.2500 mg/ml) and second highest concentrations (0.6250 mg/ml) of carvacrol resulted in significantly lower feed intake (p <0.05) with the greatest reduction of 24% feed intake at the highest concentration.

For naringenin, the regression line for feed intake vs. log of the concentration was also significant (R^2^ = 0.246, p <0.0001, y = 16.389 x^2^ − 63.799x + 541.02; Fig 3c). Feed intake was significantly lower at all the concentrations (p <0.05). However, even the greatest impact on feed intake was only a 22% reduction in feed intake with an intermediate concentration of naringenin (0.2083 mg/ml).

### Honey bee longevity with sulforaphane and naringenin

**Fig 4:**
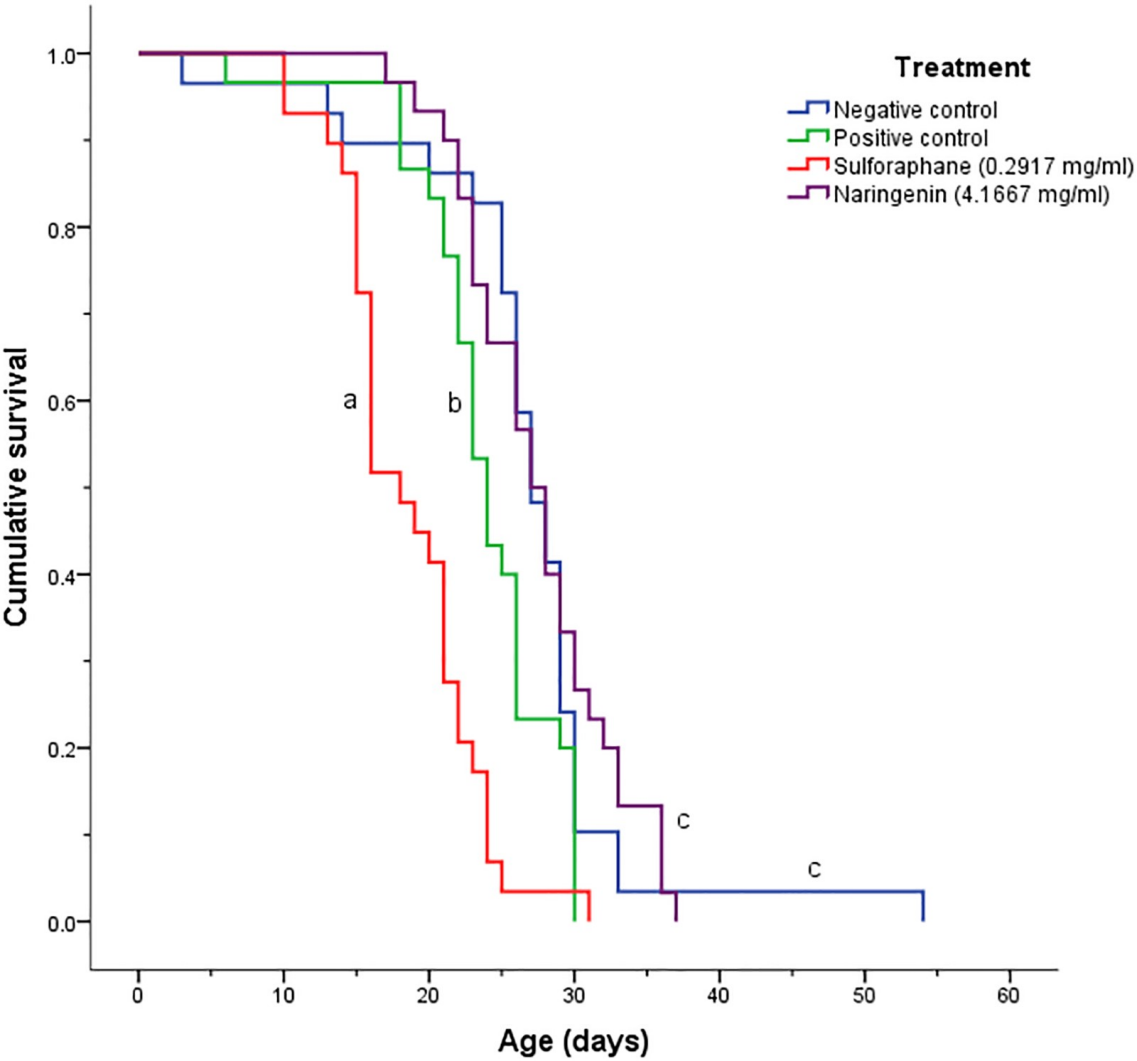
Kaplan-Meier survival curves for *N*. *ceranae*-infected honey bees fed the nutraceuticals sulforaphane and naringenin. A log rank/Mantel-Cox post hoc test was used to determine which curves were significantly different from each other (α = 0.05). Curves labelled with the same letter are not significantly different.

Kaplan-Meier survival curves for the negative control, positive control, 0.2917 mg/ml sulforaphane and 4.1667 mg/ml naringenin treatments showed significant differences using the log-rank/Mantel-Cox post hoc test (χ^2^_3_ = 52.502, p < 0.00001; Fig 4). Bees fed sulforaphane had the lowest survival, and the survival curve for sulforaphane-fed bees was significantly different from all other treatments. The next lowest survival was for positive control bees, which were also significantly different from all other treatments. The negative control bees and bees fed naringenin had the highest survival, with their survival curves not being significantly different from one another, although they were significantly different from the positive control and sulforaphane survival curves.

## Discussion

This study examined nutraceuticals and immuno-stimulatory compounds for their ability to reduce spore multiplication and reduce mortality during *N*. *ceranae* infection in honey bees. Among the compounds tested, only thymol had been previously reported to reduce *N*. *ceranae* and *N*. *apis* spore loads and reduce mortality in infected bees [38]. Of the twelve compounds tested, only hydroxytyrosol and trans-cinnamaldehyde did not show a significant effect on spore counts, indicating that many nutraceutical and immuno-stimulatory compounds may be able to control *N*. *ceranae* to varying extents.

Sulforaphane was considered to be sufficiently promising in the primary screening, and so it was also tested at several concentrations. It could reduce spore counts at all except the lowest concentration (0.0125 mg/ml) and was able to completely eliminate spores from the samples at the highest concentration tested (1.2500 mg/ml), indicating high effectiveness. Even at intermediate concentrations (0.1250 mg/ml and 0.1667 mg/ml), sulforaphane was able to reduce spore counts by 71% and 64%, respectively, compared to the positive control. One reason for this is that sulforaphane can arrest the growth of a wide range of bacteria and fungi by reducing cell proliferation and repair, and inducing apoptosis in microorganisms [25, 57].

Johansson et al. [25] noted that sulforaphane’s antimicrobial effect is likely not selective to pathogens but could also arrest growth of beneficial microbes in the gut, which would be harmful to the bee. The loss of beneficial microbes in the bee gut could have contributed to the increased mortality in the bees. Sulforaphane could also harm the bee by inducing apoptosis in host cells [25, 57]. *N*. *ceranae* infection alters expression of host genes related to apoptosis [58], with apoptosis being almost completely absent in the midgut of infected bees, likely as a means of suppressing defense responses [59]. Induction of apoptosis in the midgut by sulforaphane might cause damage to intestinal function, contributing to the increase in mortality and decrease in feed intake that was observed.

Another reason why sulforaphane could have reduced the parasite’s infection is that it is a potent inducer of antioxidant genes [60]. Infection with *N*. *ceranae* increases reactive oxygen species (ROS) generation and decreases expression and activity of antioxidants [5]. This increases oxidative stress and damages epithelial cells, which in combination with preventing cell repair, is believed to aid the parasite in spreading and infecting new cells [3]. Sulforaphane’s induction of antioxidant gene expression may have prevented this, thus depriving the parasite of this potential advantage during infection. However, too high of expression of antioxidants can be damaging to animals and prevent cell repair [61], which may have contributed to increased bee mortality and decreased feed intake.

A further reason why sulforaphane may have been toxic to the bees is that it down-regulates a number of kinases involved in the Wnt signalling pathway, thus reducing cell proliferation and repair, which helps provide sulforaphane with its anticancer properties [61]. Dussaubat et al. [3] showed that *N*. *ceranae* infection also down-regulates many genes in the Wnt pathway, leading to decreased repair of damaged tissue in *N*. *ceranae*-infected honey bees. If infected bees treated with sulforaphane experienced down-regulation of the Wnt pathway from both *N*. *ceranae* infection and sulforaphane treatment, then cell repair may have been so reduced that the midgut epithelium may have been functioning inadequately, thus resulting in increased mortality and decreased feed intake. This would be exacerbated by the damage caused by sulforaphane’s induction of apoptosis in host cells [25, 61].

Naringenin was also considered to be sufficiently promising in the primary screening that it was further tested at several concentrations, which were all effective at reducing spore counts, although not to the extent observed with sulforaphane. Naringenin is a potent anti-inflammatory compound, with its many hydroxyl groups allowing it to act directly as an antioxidant [28]. In mice and rats, oral administration of naringenin drastically reduced intestinal and liver inflammation, respectively, by down-regulating expression of free radical-generating enzymes and pro-inflammatory cytokines, thus lowering ROS levels and preventing oxidative damage [26, 28]. This could have helped control the parasite by blocking its method of spreading from one cell to another, as described previously for sulforaphane. However, little is known about cytokines or inflammation in honey bees, and therefore, anti-inflamatory activity is solely based on literature with mammals.

While naringenin did not result in a significant decrease in mortality in the primary screening or when tested at different concentrations, it did decrease mortality in infected bees when analyzed using the Kaplan-Meier survival curves. Infected, naringenin-fed bees lived as long as the uninfected control bees. A reason for this result may be that the Kaplan-Meier survival curves involve measuring mortality up until the last bee has died, unlike the mortality test in the primary screening and dose response experiments, where mortality measurements were terminated at 16 dpi. Thus, long term decreases in bee mortality with naringenin would not have been observed as the last bees dying with naringenin treatment occurred at 37 dpi.

While naringenin’s effect on reducing spore loads was only moderate, its effect on extending longevity in infected bees could make it a potential control for *N*. *ceranae* infections. Naringenin may not be able to eliminate spores and prevent their spread completely, but it may be able to significantly reduce or even eliminate symptoms of infection, such as the energetic stress and reduction in lifespan seen in infected bees [3, 5, 11].

Naringenin’s anti-inflammatory properties may explain why naringenin-fed bees were found to live significantly longer than infected, positive control bees. The increase in ROS generation and decrease in antioxidant activity caused by *N*. *ceranae* infection may be responsible for reducing the life span of infected bees [3–5]. The antioxidant activity of naringenin and its ability to down-regulate free radical-generating enzymes may have reduced these negative impacts, allowing for longer life spans.

Another reason why naringenin could have increased bee longevity is that it may positively affect the fat body of the bee. Assini et al. [26] found that feeding naringenin increased lipid metabolism in mice fed a high fat diet. If increased lipid metabolism occurred in honey bees, then it could have increased the activity of their fat body, which is the main site of lipid metabolism in insects. The fat body is related to longevity through its links to nutritional and metabolic pathways [58], and is also the main immunogenic organ, where expression of many immune-related genes is highest [4]. Increasing fat body development, such as by feeding bees pollen sources rich in lipids, increases expression of many immune genes, which could be beneficial [4]. However, further work is needed to determine if feeding bees naringenin affects fat body development.

Both naringenin and sulforaphane exhibit antioxidant and anti-inflammatory properties [26, 28, 60], but in this study, these compounds caused significantly different levels of bee mortality. This is likely due to naringenin being mainly an antioxidant and free-radical scavenger [28], while sulforaphane also has strong antimicrobial properties in addition to its effect on antioxidant gene expression [25, 61]. It is likely that sulforaphane’s inhibitory effects on cell proliferation and repair, and its induction of apoptosis were the dominant effects it had in this study, leading to a strong reduction in spore counts, but an associated increase in bee mortality.

Carvacrol was considered to be sufficiently promising in the primary screening that it was further tested at several concentrations. Like in the cases of naringenin and sulforaphane, several concentrations were effective at reducing spore counts. However, spore counts were relatively unchanged with increasing concentrations, unlike naringenin and sulforaphane, with an intermediate concentration (0.1000 mg/ml) resulting in the lowest spore counts.

Carvacrol had never been directly tested in honey bees for *N*. *ceranae* control, but thymol, as well as different essential oils containing carvacrol, have been tested against *N*. *ceranae*. Thymol significantly reduced spore counts in *N*. *ceranae*-infected bees in previous studies [37–39]. However, Maistrello et al. [37] and Costa et al. [38] did not see any significant effects until 25 dpi, while van den Heever et al. [39] saw a 40% reduction in *N*. *ceranae* spores after 17 days. Thymol and carvacrol showed a reduction in spore counts by 16 dpi in this study. Bogdan et al. [36] found that the essential oil of thyme and winter savory (both containing carvacrol and thymol) did not reduce spore counts of *N*. *apis* in honey bees in either the field or in the lab, but they did lower bee mortality associated with *N*. *apis* infection. However, the current study may be the first direct evidence that carvacrol is also effective in reducing *N*. *ceranae* spore counts.

Oregano oil was likely effective in reducing the number of spores/bee in this study because it contains carvacrol and thymol, both having activity against bacterial and fungal pathogens [49, 50, 62]. However, it may be less desirable as a treatment compared to purified thymol or carvacrol as the amount of active ingredients cannot be controlled in a plant extract. For example, carvacrol levels in oregano oil can vary between 50 and 70% [54]. Like carvacrol and thymol, oregano oil did not affect bee mortality in the primary screening, although oregano oil has been shown to have a slightly toxic effect on bees [54, 55].

Tetrahydrocurcumin and embelin caused reductions in spore counts in this study. Tetrahydrocurcumin and embelin are potent anti-inflammatories when fed to mice and significantly reduce oxidative damage [31, 63, 64]. Tetrahydrocurcumin is a metabolite of curcumin, the main active ingredient in the spice turmeric (*Curcuma longa*) [63], and curcumin reduced *Nosema* spp. spore loads and increased survival and overall health of infected bees [40]. Embelin and tetrahydrocurcumin also have stimulatory effects on lipid metabolism [32, 63], similar to that of naringenin [26] and carvacrol [23], which could increase the immune response associated with the fat body of the bee as previously described [4].

Allyl sulfide in this study reduced spore counts, but did not affect mortality. Ally sulfide, allicin, and other garlic components possess strong antimicrobial properties, with garlic extracts reducing infection prevalence of *Nosema bombycis* in the silkworm, *B*. *mori* [20], and allicin inhibiting growth of the honey bee pathogens, *Paenibacillus larvae* and *Ascosphaera apis* [65]. However, Porrini et al. [66] found that garlic extracts were not effective in the control of *N*. *ceranae* infection in honey bees. The authors pointed out that while allicin and its metabolites are antimicrobial, they are extremely unstable, especially in ethanolic extracts. However, isolated allyl sulfide did reduce spore counts in this study, perhaps because it is more antimicrobial or somewhat more stable than other ethanolic garlic extracts.

Poly I:C also reduced *N*. *ceranae* spore counts in this study. It is a synthetic, double-stranded viral RNA molecule that acts as an immunostimulant in mammals and birds by binding to the immune Toll-like receptor TLR3 and initiating an immune response [33, 53]. While it is unknown if insects possess an exact TLR3 homolog, injection of poly I:C activates the Toll immune pathway in an arthropod, the Pacific white shrimp, *L*. *vannamei* [34]. The effectiveness of poly I:C in this study could have been due to it activating the Toll immune pathway, which has been related to resistance to *N*. *ceranae* in honey bees [9]. However, Parvizi et al. [33] and Li et al. [34] injected poly I:C, thus avoiding any possible degradation in the midgut, unlike this study where it was fed to the bees.

The chitin-containing polysaccharide chitosan reduced *N*. *ceranae* spore counts in this study. It can act as a fungal pathogen-associated molecular pattern (PAMP), inducing a strong immune response when fed to chickens [35]. In addition, feeding chitosan to honey bees also induced a strong immune response by increasing expression of a number of AMP genes as well as expression of the immune and longevity-related gene, *vitellogenin* [36]. Chitosan is also a weak antimicrobial compound, though it is mainly effective against bacteria [67, 68]. Thus, it is not clear if the reduced spore counts are more due to an increased immune response or an antimicrobial effect.

In this study, almost all the compounds tested showed some promise as alternative controls for *N*. *ceranae* infection in *A*. *mellifera*. However, the level of control was always limited and not comparable to the effectiveness of fumagillin, as none of the compounds–with the exception of sulforaphane–were able to completely eliminate spores. Sulforaphane was particularly effective, but more research is needed on ways of reducing its high toxicity to bees. While naringenin significantly reduced spore counts, it was most notable and promising for its effect on increasing longevity of infected bees. More work is needed to determine if even higher concentrations may be more effective without affecting mortality. However, while lower concentrations of naringenin similarly increased the longevity of *Drosophila melanogaster*, higher concentrations were lethal [69]. Thus there may actually be detrimental effects to *A*. *mellifera* with high concentrations of naringenin. If the mode of action of naringenin, carvacrol, embelin and tetrahydrocurcumin in honey bees is related to the fat body, then their effectiveness could be increased if the compounds were fed in combination with a lipid-rich diet, which may also increase fat body development and immune function. This research has identified several promising compounds, but work is needed to understand their mode of action to achieve economically viable effectiveness.
